# A Novel Platform for the Potentiation of Therapeutic Antibodies Based on Antigen-Dependent Formation of IgG Hexamers at the Cell Surface

**DOI:** 10.1371/journal.pbio.1002344

**Published:** 2016-01-06

**Authors:** Rob N. de Jong, Frank J. Beurskens, Sandra Verploegen, Kristin Strumane, Muriel D. van Kampen, Marleen Voorhorst, Wendy Horstman, Patrick J. Engelberts, Simone C. Oostindie, Guanbo Wang, Albert J. R. Heck, Janine Schuurman, Paul W. H. I. Parren

**Affiliations:** 1 Genmab, Utrecht, The Netherlands; 2 Biomolecular Mass Spectrometry and Proteomics, Bijvoet Center for Biomolecular Research and Utrecht Institute for Pharmaceutical Sciences, Utrecht University, Utrecht, The Netherlands; 3 Netherlands Proteomics Centre, Utrecht, The Netherlands; 4 Department of Cancer and Inflammation Research, Institute of Molecular Medicine, University of Southern Denmark, Odense, Denmark; 5 Department of Immunohematology and Blood Transfusion, Leiden University Medical Center, Leiden, The Netherlands; Scripps Research Institute, UNITED STATES

## Abstract

IgG antibodies can organize into ordered hexamers on cell surfaces after binding their antigen. These hexamers bind the first component of complement C1 inducing complement-dependent target cell killing. Here, we translated this natural concept into a novel technology platform (HexaBody technology) for therapeutic antibody potentiation. We identified mutations that enhanced hexamer formation and complement activation by IgG1 antibodies against a range of targets on cells from hematological and solid tumor indications. IgG1 backbones with preferred mutations E345K or E430G conveyed a strong ability to induce conditional complement-dependent cytotoxicity (CDC) of cell lines and chronic lymphocytic leukemia (CLL) patient tumor cells, while retaining regular pharmacokinetics and biopharmaceutical developability. Both mutations potently enhanced CDC- and antibody-dependent cellular cytotoxicity (ADCC) of a type II CD20 antibody that was ineffective in complement activation, while retaining its ability to induce apoptosis. The identified IgG1 Fc backbones provide a novel platform for the generation of therapeutics with enhanced effector functions that only become activated upon binding to target cell–expressed antigen.

## Abstract 2

Efficient activation of the complement system naturally involves hexamerization of antibodies upon target binding at the cell surface. Screening a library of IgG-Fc mutants reveals how to enhance the effector functions of therapeutic antibodies by maximizing hexamer formation.

## Author Summary

Immunotherapy is a powerful and rapidly expanding field that makes use of the body’s natural defense mechanisms to eliminate disease entities such as infectious agents or cancer cells. Circulating antibodies bind aberrant structures in a highly target-specific manner and “flag” disease cells for destruction by killing machineries that are present in the bloodstream. We recently showed that activation of one of these immune defense mechanisms, the complement system, is most efficiently initiated by binding of the first complement component C1q to a ring of six antibodies. Since antibody hexamerization occurs naturally only after binding to surface antigens, complement activation and subsequent complement-mediated cell killing is therefore restricted to these antibody-flagged cells. Now, with a mutational screening approach, we identified structural entities in the antibody backbone that potentiate this antigen binding–induced hexamer formation. We identified mutations that enhance the hexamer formation and complement activation by IgG1 antibodies against a wide range of targets on varying cancer cells. Based on our findings, we present a broadly applicable platform for the generation of therapeutic antibodies with enhanced ability to promote hexamerization-induced complement activation and target cell killing only after surface antigen binding.

## Introduction

Target cells are flagged for destruction by antibodies bound to their cognate antigen on the cell surface. Elimination of antibody-opsonized cells is mediated by the innate immune system. The cellular branch of this system includes NK cells, monocytes, macrophages, and neutrophils that are activated via specific IgG Fc receptors (FcγR) sensing surface-bound IgG antibodies. The molecular branch of innate defense includes the complement system, which consists of an amplifiable cascade of soluble zymogens that are abundant in blood and other extracellular fluids. We recently showed that IgG antibodies organize into ordered hexamers on cell surfaces following antigen binding. These IgG hexamers bind and activate C1, the first component in the classical complement pathway that leads to target cell killing by complement-dependent cytotoxicity (CDC) via membrane attack complexes (MACs) that breach the cell membrane [1]. In addition, complement activation generates chemoattractants, anaphylatoxins, and opsonins that serve to attract and activate immune effector cells and induce additional killing [2]. In immunotherapy, we leverage these natural defense mechanisms by marking specific target cell populations for elimination by passively administered therapeutic antibodies. These antibodies may be engineered to enhance their ability to activate effector cells or complement. For example, amino acid residues in IgG that affect binding in an FcγR-specific fashion can be modified to promote more efficient antibody-dependent cellular cytotoxicity (ADCC) or antibody-dependent cellular phagocytosis [3–5]. C1 binding and CDC may be increased by reshuffling IgG1 and IgG3 or by mutating amino acid positions adjacent to the lower hinge [6–11]. In contrast to IgG molecules, IgM antibodies already pre-exist as pentameric or hexameric oligomers that are kept together via covalent bonds. Exposure of the C1 binding site and the activation of complement is regulated via conformational changes upon antigen binding [12,13]. This concept to enhance complement activation has been exploited by the covalent association of IgG monomers via disulfide bonds between cysteine residues in an IgM-derived 18 amino acid carboxyterminal extension and additionally between cysteine residues introduced at position 309 [14].

The ability and potency of monoclonal antibodies (mAbs) to induce complement activation and CDC is dependent on IgG isotype and on the characteristics of both the antigen (i.e., size, flexibility, and mobility) and the epitope (i.e., accessibility and distance to the membrane). The potential of complement for rapid and effective cell killing, as well as its capacity for attracting and modulating both innate and adaptive cellular immune responses, provide a strong rationale for the development of therapeutic antibodies with optimized complement-activating characteristics [2]. However, few therapeutic antibodies generated to date induce potent CDC [2,15–17], and the availability of technologies to facilitate the generation and development of IgG antibodies that are capable of effective complement activation are therefore highly sought after.

We recently demonstrated that the assembly of IgG molecules into hexamers may be enhanced by specific mutations in the Fc domain [1]. Here, we identify two preferred IgG1 backbones, harboring a single amino acid mutation, that strongly enhance Fc-mediated assembly at the cell surface, while retaining all the qualities required for the development of biopharmaceuticals. We demonstrate applicability to a range of targets and cells including hematological and solid tumor indications as well as cell lines and patients’ cells. The conditional nature of the technology provides a novel route for the identification and safe development of potentiated Fc-based immunotherapeutics.

## Results

### Potentiation of CDC Activity by Increased Hexamerization Is Broadly Applicable

Previously, we showed that antigen-bound IgG molecules organize into hexamers at the cell surface to mediate optimal C1q binding and complement activation. We observed an extensive Fc:Fc interface between IgG molecules, and it was shown that a glutamic acid into arginine (E345R) mutation at this interface enhanced IgG clustering, C1q binding, and CDC [1]. To determine whether this observation could be broadly applied, we introduced the E345R mutation in a panel of IgG antibodies targeting different antigens (CD20 [Uniprot P11836], CD52 [P31358], CD38 [P28907], and epidermal growth factor receptor [EGFR] [P00533]) and assessed CDC of cell lines with varying expression of antigen and membrane complement regulatory proteins (mCRPs) CD46 (P15529), CD55/DAF (P08174), and CD59 (P13987).

**Fig 1:**
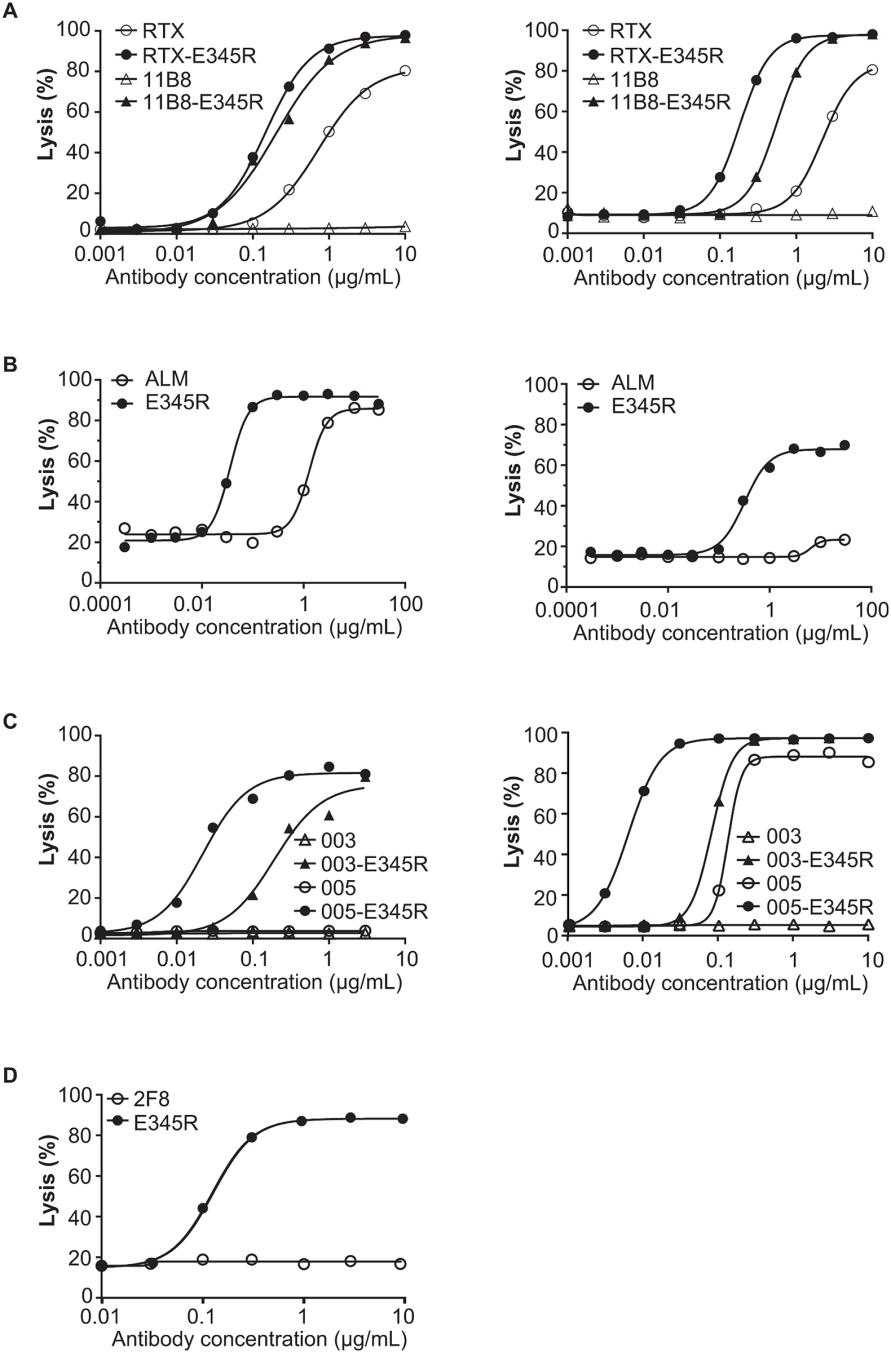
CDC enhancement by promoting Fc:Fc interactions is broadly applicable. A representative example of three replicates is shown. Each graph compares CDC in dose response for the wild-type and E345R-mutated mAb. (A) CDC of Wien 133 (left) and Daudi cells (right) opsonized with anti-CD20 mAbs RTX and 11B8. (B) CDC of Wien 133 (left) and Raji cells (right) opsonized with anti-CD52 mAb ALM. (C) CDC of Wien 133 cells (left) and Daudi cells (right) opsonized with anti-CD38 mAbs IgG1-003 and IgG1-005. (D) CDC assay of A431 cells opsonized with anti-EGFR mAb 2F8.

Introduction of the E345R mutation into the chimeric CD20 antibody rituximab (RTX) as well as the human CD20 antibody 11B8 increased lysis of both Wien 133 and Daudi cells (Fig 1, S1 Table). The difference was particularly apparent for wild-type 11B8, which, as expected for a type II CD20 antibody, did not induce complement-mediated lysis (Fig 1A). Introducing E345R in the CD52 antibody alemtuzumab (ALM) strongly enhanced CDC of both Wien 133 and Raji cells (Fig 1B), and the mutation also potentiated both the CD38 antibodies IgG1-005 and IgG1-003 (Fig 1C). Interestingly, antibody 2F8 directed against the solid-tumor antigen EGFR also induced potent CDC when containing E345R (Fig 1D). IgG1-003 and 2F8 represent antibodies that were unable to induce CDC in previous studies [18,19]. From these data, we conclude that the Fc:Fc interface mutation E345R is able to convert antibodies against a range of targets and epitopes into potent mediators of CDC, even for antibodies that do not induce any complement-mediated lysis as a wild-type IgG1 molecule.

**Fig 2:**
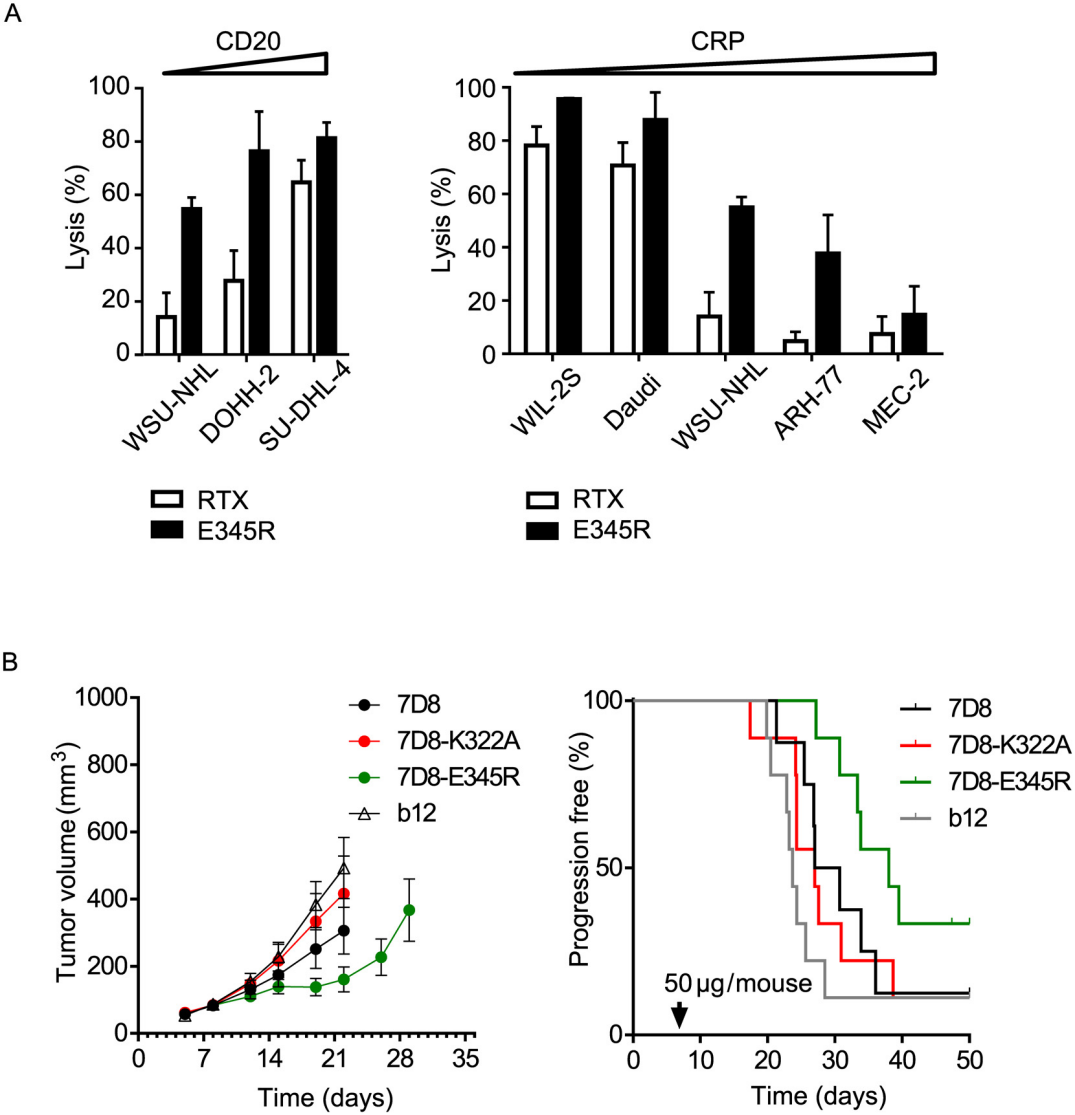
Promoting Fc:Fc interactions alleviates CDC sensitivity to target and mCRP expression. (A) Cell lines were opsonized with saturating amounts of wild-type or mutated RTX and analyzed by CDC assay. Left panel: cell lines with increasing CD20:mCRP ratio (left to right); right panel: cell lines with decreasing CD20:mCRP ratio (left to right). (B) In vivo analysis of subcutaneous tumor growth in a severe combined immunodeficiency (SCID) xenograft model. SCID mice were injected with luciferase-expressing Raji cells. Eight days after tumor cell injection, mice were randomized at an average tumor volume of 85 mm^3^ per group and treated with 50 μg IgG1 antibody. Tumor volumes were monitored over time and depicted as average tumor volume ± standard error of the mean (SEM) (left panel), and as Kaplan-Meier curves with time to progression cutoff set at a tumor volume of >700 mm^3^ (right panel).

To analyze the impact of target expression, we performed CDC assays using a panel of human tumor cell lines with increasing CD20 expression. Cells were selected for a comparable (intermediate) expression of total mCRPs resulting in increasing CD20:mCRP ratios (S2 Table). Only highly CD20-overexpressing SU-DHL-4 cells were lysed efficiently by RTX, whereas lower expression resulted in minimal lysis. In contrast, introduction of E345R in RTX yielded strong lysis for all three cell lines tested (Fig 2A). The potential of mCRPs to limit CDC by wild-type and E345R-mutated RTX was assessed with cell lines that express similar levels of the CD20 target antigen and increasing levels of mCRPs, resulting in decreasing CD20:mCRP ratios (Fig 2A, S2 Table). RTX-E345R demonstrated increased lysis for all cell lines tested, indicating that the mutation reduced sensitivity to mCRP inhibition.

The in vivo efficacy of E345R-potentiated IgG1 antibody 7D8, directed against CD20, was evaluated in severe combined immunodeficiency (SCID) mice inoculated subcutaneously with luciferase-expressing Raji cells expressing high numbers of complement defense molecules (Fig 2B). After the tumor volume reached approximately 40–150 mm^3^, mice were randomized at an average tumor volume of 85 mm^3^ per group and treated intraperitoneally with a single dose of 50 μg 7D8, 7D8-E345R, 7D8-K322A, or an irrelevant IgG1 isotype control antibody (IgG1-b12). 7D8-K322A contains a mutation that abrogates C1q binding and CDC and was used as an additional control. In agreement with its increased ability to activate complement, only 7D8-E345R showed significant antitumor efficacy. The wild-type 7D8 showed a trend of activity, but did not reach statistical significance compared to controls. Statistical analysis of tumor inhibition was performed on day 22 when all mice in all groups were still in the study (S3 Table). 7D8-E345R inhibited tumor growth vs control (*p* = 0.0044) and compared to 7D8-K322A (*p* = 0.0474). In addition, in a Kaplan Meier analysis performed until the end of the experiment on day 44 (S3 Table and Fig 2B), 7D8-E345R significantly inhibited progression as defined by tumors growing > 700 mm^3^ (*p* = 0.019), whereas the wild type did not.

### Library Screening Demonstrates All Mutations of E345 and E430 Stimulate CDC

**Fig 3:**
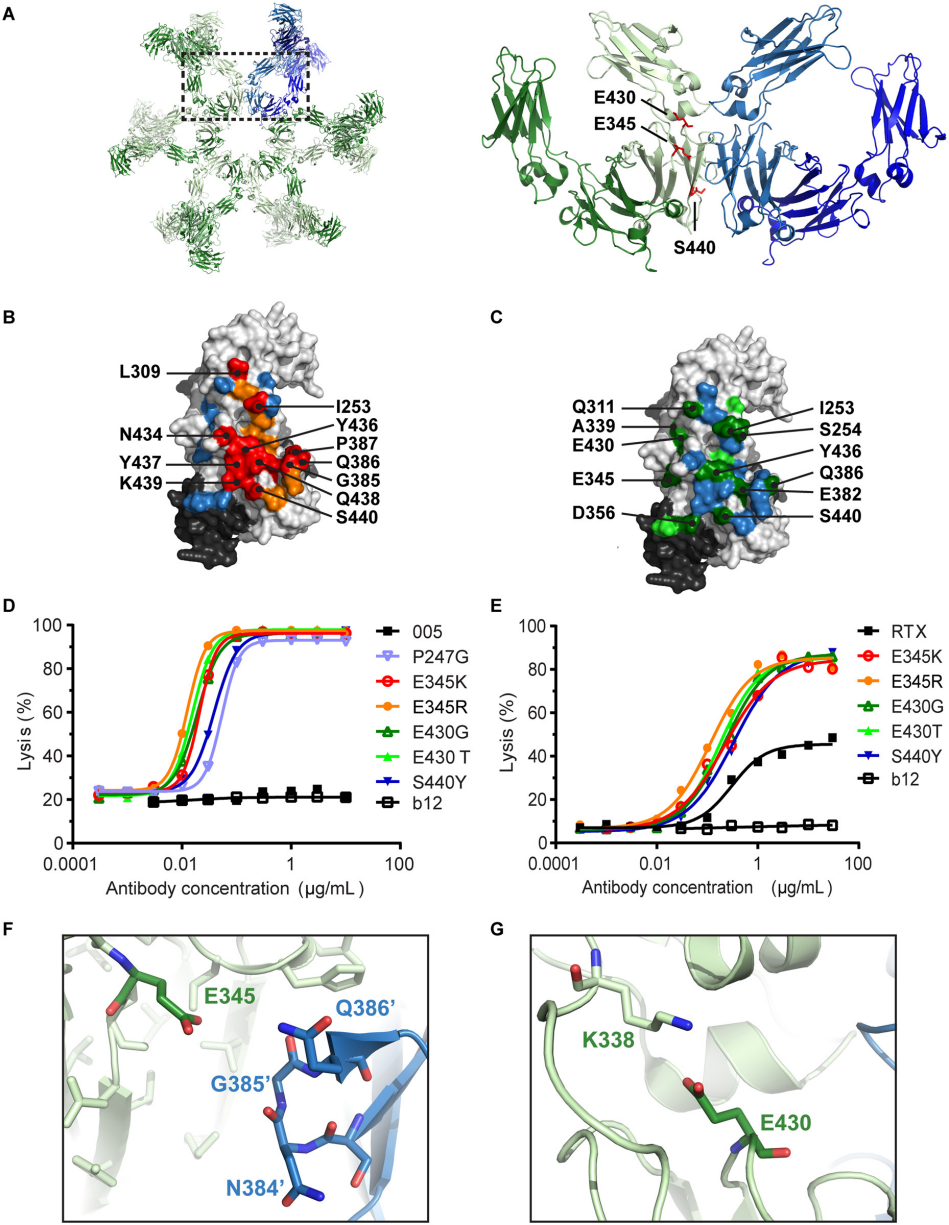
E345 and E430 limit Fc:Fc interactions. (A) Left: overview of the IgG1 antibody hexamer observed in the IgG1-b12 crystal structure (PDB access code 1HZH [20]). Right: zoomed-in view of two neighboring Fc domains with enhancing amino acid positions highlighted. (B) Summary of CDC inhibition data. The surface view is rotated 90° relative to panel A to show the Fc:Fc interface. Daudi cells were opsonized with mutants of anti-CD38 mAb IgG1-005 (1.0 μg/mL) and assessed for CDC. Light and dark grey colors indicate the unmodified amino acids of the two heavy chain regions composing a single Fc domain. All mutants assessed were ranked by efficiency per amino acid position, and the resulting median lysis efficacy is indicated by coloring per position; orange and red colors indicate positions with reduced lysis. Blue: >60% lysis; orange: 30%–60% lysis; red: <30% lysis. (C) Summary of CDC enhancement data. Wien 133 cells were opsonized with mutants of anti-CD38 mAb IgG1-005 (1.0 μg/mL) and assessed for CDC. Light and dark grey colors indicate unmodified amino acids as described under (B). The maximal lysis of the most efficient mutant per amino acid position is displayed with green indicating positions for which CDC was enhanced. Blue: <20% lysis; light green: 20%–40% lysis; dark green: >40% lysis. (D) Dose response in CDC of Wien 133 cells by IgG1-005 and selected mutants. A representative of 3 replicate experiments is shown. (E) Dose response in CDC of Ramos cells induced by RTX and selected mutants. A representative experiment of 3 replicates is shown. (F) Structural view zoomed to residue E345. Amino acids facing E345 at the Fc:Fc interface are indicated in blue and named with apostrophe. (G) Structural view zoomed to residue E430, forming a salt bridge with K338 at the intramolecular C_H_2–C_H_3 domain interface.

To better understand the structural constraints of IgG hexamerization and to identify mutations other than E345R that promote Fc:Fc interactions, we generated an antibody mutant library focused on two regions of interest. Firstly, amino acid substitutions were introduced at, or proximal to, the intermolecular Fc:Fc interface observed in the hexameric crystal packing of human IgG1 [1,20]. Secondly, amino acid substitutions were introduced at the intramolecular C_H_2–C_H_3 interface to alter Fc domain flexibility, which could potentially modulate Fc:Fc interactions allosterically (Fig 3A). We employed screening conditions that allowed for the identification of inhibitory and stimulatory mutations. Mutants of CD38 antibody IgG1-005 containing single amino acid mutations were screened with the complement-sensitive cell line Daudi at a concentration that induced maximal lysis for the wild-type antibody to identify inhibitory positions (Fig 3B; S4 Table). Stimulatory mutations were screened for using the complement-refractory cell line Wien 133 at a concentration that induced minimal CDC for the wild-type antibody (Fig 3C, S5 Table).

As shown by the orange- and red-colored residues in Fig 3B and S4 Table, most mutations at the Fc:Fc interface inhibited CDC, corroborating our previous observations [1] and confirming that Fc:Fc interactions are a prerequisite for efficient complement activation. The consensus IgG interaction site for FcRn, Protein A, and Protein G (S1A Fig) [21] was found to play a crucial role in the interaction between antibody Fc domains, as exemplified by the abundance of CDC-inhibiting mutations in this region (S1B Fig). The C-terminal β-strand represents another critical region as mutations of all amino acids in the stretch 437–440 inhibited CDC, demonstrating it provides important contacts for an efficient Fc:Fc interaction (S1C Fig).

Mutations promoting Fc:Fc interactions were relatively sparse and predominantly confined to the periphery of direct Fc:Fc contacts as indicated by the green colored residues in Fig 3C and S5 Table. A selection of the stimulating mutations were separately introduced in RTX and, next to the IgG1-005 mutants, assessed in dose response. This analysis demonstrated that the E345, E430, and S440 mutations that enhanced CDC of Wien 133 cells by IgG1-005 (Fig 3D) also consistently stimulated CDC of Ramos cells by RTX (Fig 3E) and Daudi cells by IgG1-005 and RTX variants (S2 Fig, S6 and S7 Tables).

The observation that all mutations at amino acid positions E345 and E430 stimulate CDC is surprising (S5 Table). A close-up of the structure surrounding the E345 Fc:Fc interface residue shows that its side chain is directed towards residue G385 at the terminus of C_H_3 β-strand C of the facing antibody (Fig 3F), according to the Ig fold nomenclature of Halaby [22]. Residue E430 forms a salt bridge stabilizing the C_H_2–C_H_3 interface packing (Fig 3G). Since even conservative mutations into aspartic acid, preserving the negative charge, resulted in enhanced CDC, glutamic acid residues at these positions appear to restrain IgG molecules in a conformation that inhibits Fc:Fc interactions and CDC. In summary, a number of CDC-enhancing point mutations were identified, with amino acids E345 and E430 acting as hotspots controlling antibody hexamerization and complement activation.

### Solution-Phase Complement Activation and Pharmacokinetics

**Fig 4:**
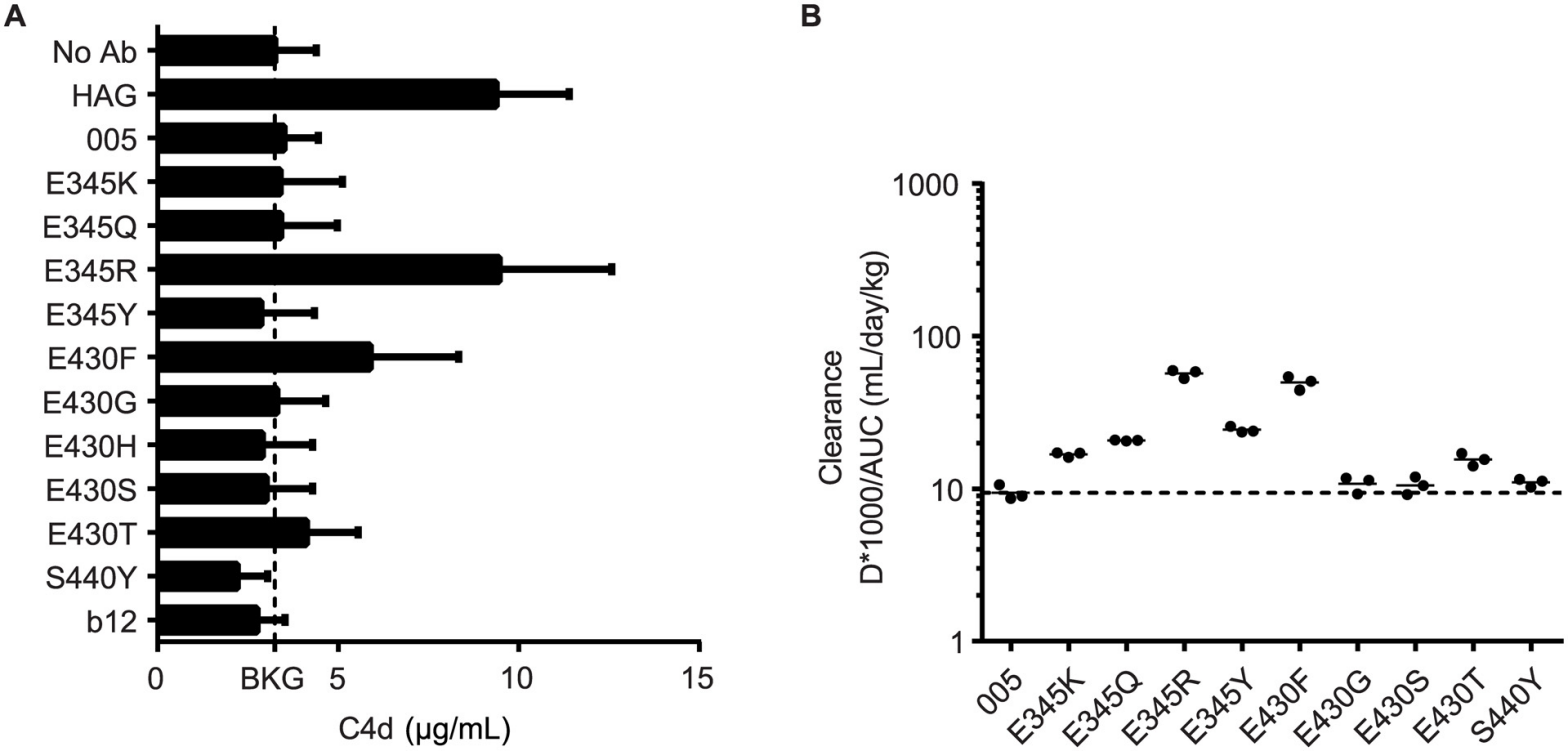
Specific IgG mutations retain fluid phase complement suppression and regular pharmacokinetics. (A) C4d ELISA of IgG1-005 mutants detecting solution-phase complement activation in serum. The background (BKG) C4d level in serum is indicated with a dashed line. Results were averaged over three experiments. (B) PK analysis of IgG1-005 mutants in SCID mice, using three mice per group. Clearance of a single 500 μg dose of mAb was monitored for three weeks and is expressed as 1,000 * Dose / area under the curve (AUC); dashed line: clearance of wild-type IgG1-005.

For therapeutic application of the mutants, nonspecific complement activation in the extracellular fluid is undesired and hexamerization should therefore only occur after the antibody binds its target at the cell surface. We therefore incubated the panel of mutants in human serum (90%) at a high, 100 μg/ml, concentration, and we assessed the generation of complement activation product C4d (Fig 4A). C4d generation by heat aggregated IgG (HAG) was used as a positive control. Complement activation, indicative of hexamer formation in solution, was only observed for mutants E345R and E430F, whereas all others did not induce C4d generation.

To fully exclude hexamerization in vivo, we used pharmacokinetic analyses in C.B-17 SCID mice as an additional assessment. Larger molecular species of IgG, potentially interacting with C1, would be expected to clear more rapidly than monomeric IgG molecules. The observed clearance rates in mice were consistent with the observed C4d generation in human serum (Fig 4B). Whereas mutants E345R and E430F displayed somewhat faster clearance rates, the other variants displayed clearance rates similar to wild-type IgG1-005. These findings were corroborated by the pharmacokinetic behavior observed for RTX variants (S3 Fig).

### Biophysical Characterization of Antibody Hexamerization in Solution

**Fig 5:**
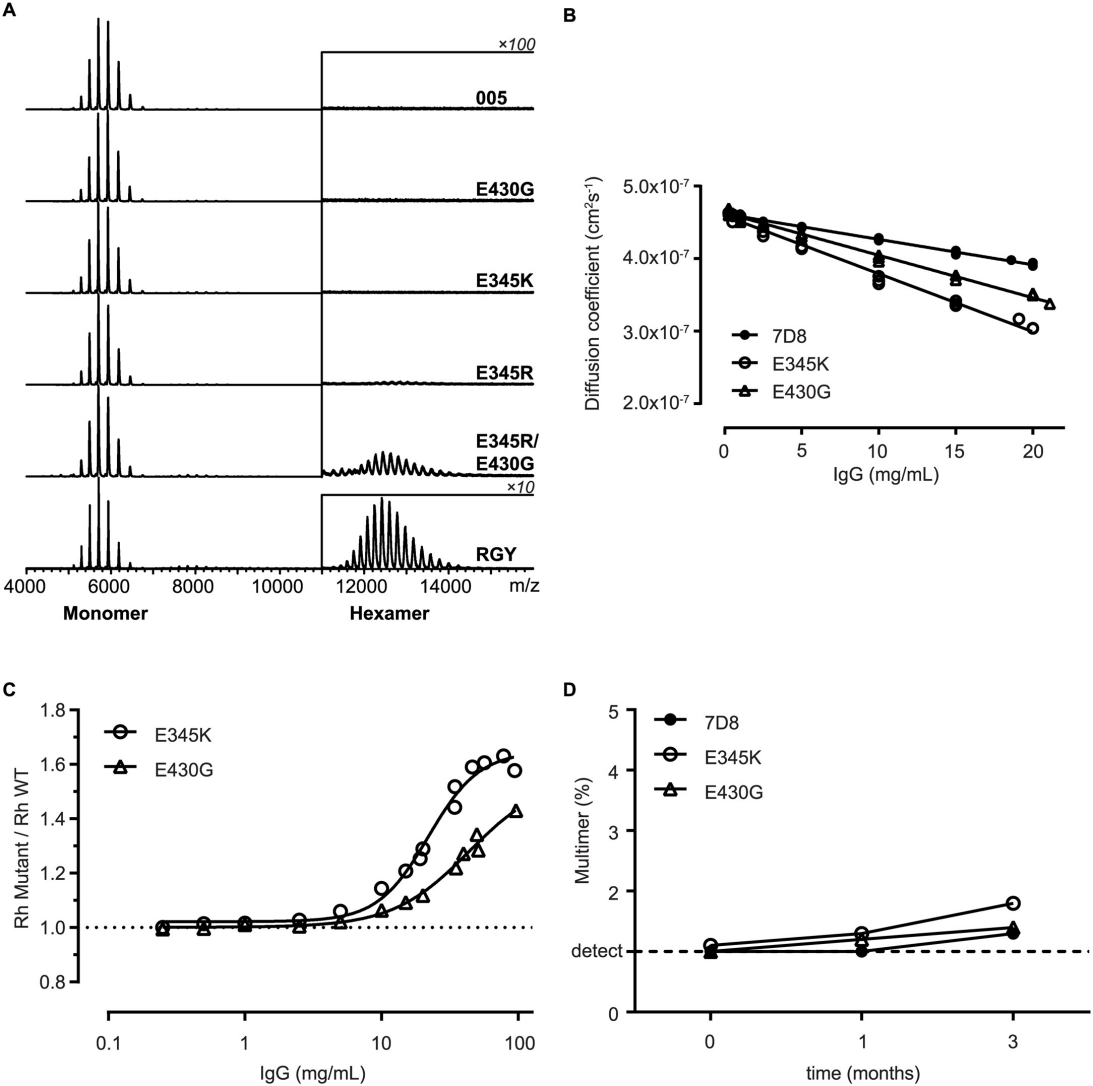
Biophysical characterization of antibody hexamerization in solution. (A) Native MS analysis of solutions of IgG1-005 and mutant antibodies at 2 μM. IgG1-005-RGY (RGY), a previously described triple mutant, is used as a positive control. The charge envelope of the IgG monomers appears around *m/z* 5,500 (~ 147 kD Mw) and that of the IgG hexamers around *m/z* 12,500 (~890 kD Mw). The signals of the hexameric species are displayed at a 100-fold or 10-fold (RGY only) amplification. (B–C) Dynamic light scattering analysis of 7D8 and mutant solutions formulated in PBS pH 7.4. Three independent experiments are shown. (B) Linear regression of diffusion coefficient versus Ab concentration. (C) Hydrodynamic radii (Rhs) of mutants measured by dynamic light scattering (DLS) were divided by Rh of wild-type 7D8 to correct for viscosity without masking the contribution of Fc:Fc self-association. (D) HP-SEC analysis of 100 mg/mL 7D8 and mutant antibody solutions formulated in PBS, incubated for three months at 5°C. Multimer detection limit of 1% is indicated by a dashed line.

To assess the suitability of the IgG mutants for their potential development as biopharmaceuticals, we analyzed their biophysical characteristics with an emphasis on methods that could detect solution-phase multimers. Firstly, we analyzed the mutants by native mass spectrometry (MS) [23]. Similar to wild-type IgG1-005, the mutants E345K and E430G only showed a charge envelope around *m/z* of 5,500, corresponding to a 147 kDa molecular weight (Mw) IgG monomer. Mutant E345R, in contrast, yielded an additional charge envelope around *m/z* of 12,500, corresponding to an 890 kDa Mw demonstrating that it forms some hexamers in solution, albeit at very low abundance (1.2% of total antibody mass) (Fig 5A). As a reference, we used the triple mutant IgG1-005-E345R/E430G/S440Y (RGY) that was previously shown to form hexamers in solution by HP-SEC, native MS and cryo electron tomography [1]. An E345R-E430G double mutant and the RGY triple mutant displayed a further increase in solution-phase hexamer formation, amounting to about 7.7% and 73% of total antibody mass, respectively. We were unable to detect single mutant species with increased Mw by high-performance size exclusion chromatography (HP-SEC) as well as multiangle laser light scattering analysis of hollow fiber flow field-flow fractionation separated fractions (S4 Fig).

To obtain sufficient material for the analysis of highly concentrated mAb solutions, we next generated three stable CHO cell lines producing 7D8 wild-type and the mutants E345K and E430G. Purified antibodies were concentrated to 100 mg/mL and analyzed by dynamic light scattering (DLS), since it is exquisitely sensitive to the presence of high molecular weight species and tolerates highly concentrated protein solutions. Regression analysis of the diffusion coefficient [24] demonstrated slightly increased attractive interactions for both the E345K and E430G mutant (−17.4 x 10^−3^ mL·g^−1^ and −12.6 x 10^−3^ mL·g^−1^, respectively) compared to wild-type 7D8 (−7.57 x 10^−3^ mL·g^−1^) (Fig 5B). At concentrations above 10 mg/ml, the Rhs of the two mutants increased more strongly than the wild-type antibody (Fig 5C). To assess whether these slightly increased self-interactions impacted the stability of highly concentrated liquid antibody formulations, 100 mg/ml formulations in PBS of 7D8 and the mutants were incubated at 5°C for three months and monitored by biophysical methods (Fig 5D, S5 Fig). The aggregation and other biophysical characteristics for both mutants could not be distinguished from the wild-type IgG1, indicating that the increased self-interactions at high concentration were fully reversible.

### Effector Functions of IgG Mutants with Enhanced Fc:Fc Interactions

**Fig 6:**
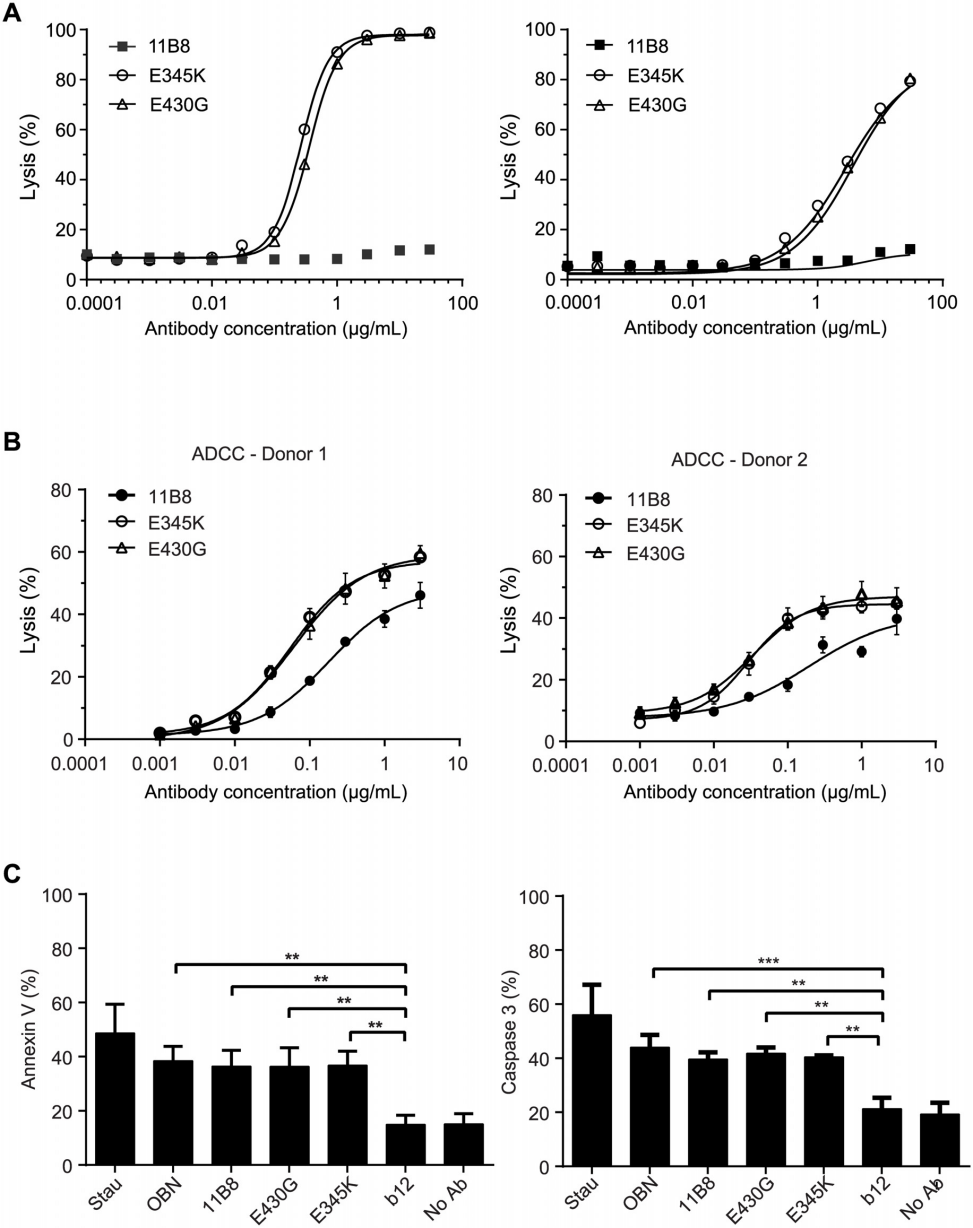
Anti-CD20 antibodies can be enhanced for CDC activity while enhancing or retaining other effector functions. (A) Daudi cells (left) or Raji cells (right) were opsonized with wild-type or mutant 11B8 and analyzed in CDC assays. A representative of three replicate experiments is shown. (B) Raji cells were opsonized with wild-type or mutant 11B8 and analyzed in ADCC assays using PBMCs isolated from two individual donors (donor 1, FcγRIIa H/H & FcγRIIIa V/V [14% NK cells]; donor 2, FcγRIIa R/R & FcγRIIIa V/F, [8% NK cells]). Two representative examples of five donors are shown. (C) Daudi cells were opsonized with wild-type or mutant 11B8 and assessed for PCD by (left) Annexin V staining and (right) cleaved Caspase 3. 11B8, 11B8-345K, 11B8-E430G and OBN are significantly different from IgG1-b12 (three replicate experiments, one-way Anova *p* < 0.05).

The potential of the IgG1-E430G and IgG1-E345K mutants was further assessed by studying anti-CD20 antibody 11B8, for which we could address the impact of hexamerization-enhancing mutations on CDC, ADCC, as well as the induction of programmed cell death (PCD). The type II CD20 antibody 11B8 induces ADCC and PCD (in the absence of exogenous crosslinking), but not CDC. Consistent with the data above, both the E345K- and the E430G-mutated 11B8 induced potent CDC, whereas the wild-type antibody did not (Fig 6A). Interestingly, the ability of 11B8 to induce ADCC was also significantly improved by both mutations (Fig 6B). To study PCD induction by 11B8, Daudi cells were stained for Annexin V (P08758) positivity and active, cleaved Caspase 3 (P42574, Fig 6C). The magnitude of PCD was similar to that induced by the wild-type 11B8 antibody and to obinutuzumab (OBN) that was used as a positive control. In summary, both the E430G- and E345K-mutated IgG1 displayed increased CDC and ADCC, while retaining the type II CD20 antibody-specific ability to induce PCD.

### Efficient Lysis of CLL Patient Tumor Cells

**Fig 7:**
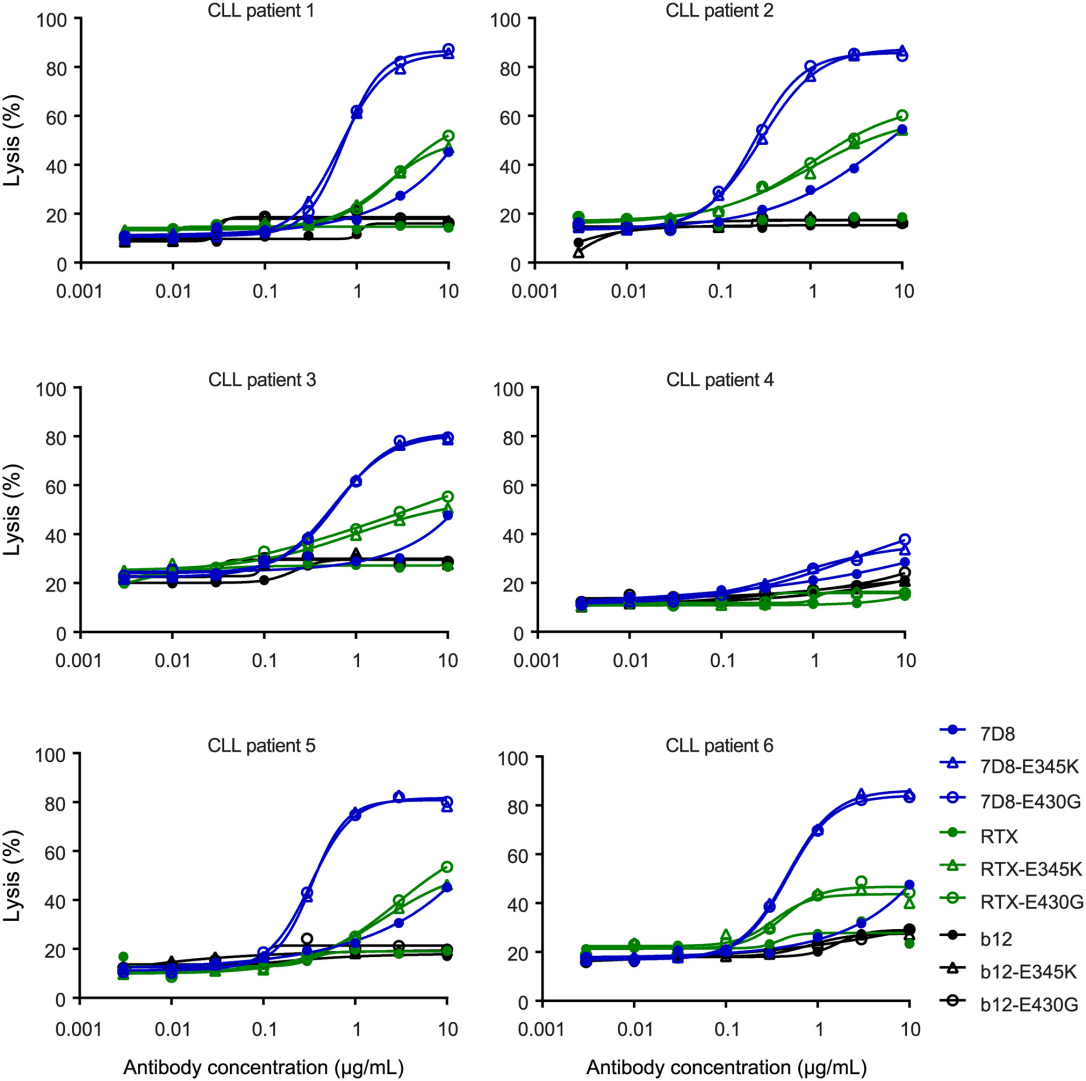
Efficient lysis of CLL patient tumor cells. Peripheral B cells isolated from the blood of six CLL patients were opsonized with wild-type or mutant, 7D8, RTX or control (IgG1-b12) antibody and tested in CDC assays. The CLL cells of patient 4 were highly refractory to CDC due to their very low CD20 expression.

Based on their strong enhancement of antigen-dependent CDC, favorable biophysical characteristics, stability, monomericity, absence of solution-phase complement activation, regular pharmacokinetics, and retained effector functions, we selected IgG1-E430G and IgG1-E345K as the preferred mutants. The therapeutic potential of these mutant IgGs was assessed in ex vivo CDC assays with tumor cells obtained from patients with chronic lymphocytic leukemia (CLL). Patient CLL cells usually are relatively resistant to anti-CD20-induced CDC due to their low CD20 and high mCRP expression [16]. Interestingly, the E345K and E430G mutations, introduced into CD20 mAb 7D8, provided strongly enhanced CDC of patient cells (Fig 7). Moreover, both mutations enabled killing of 5 out of 6 patient CLL cells by RTX, where wild-type RTX was unable to induce CDC.

## Discussion

Activation of complement factor C1 by IgG antibodies via the classical pathway requires antigen binding and hexamerization of the antibody at the cell surface. Here, we show that the ability of IgG1 antibodies to form hexamers can be enhanced by single point mutations at specific amino acid positions both at the intermolecular Fc:Fc and the intramolecular C_H_2–C_H_3 interface. Several IgG1 mutants, exemplified by E430G and E345K, displayed strongly enhanced CDC that remained conditional on antigen binding at the target cell. Increased self-association of these mutants was only apparent at high (10–100 mg/mL) concentrations in solution. This self-association was fully reversible and did not result in the formation of aggregates. Furthermore, the slight increase in self-association for these mutants did not lead to solution-phase complement activation in serum or accelerated pharmacokinetics.

Mutations of the Fc:Fc interface residue E345 yielded potent activation of CDC irrespective of the amino acid substitution. It therefore appears unlikely that mutation-specific interactions drive hexamerization. We hypothesized that the wild-type E345 side chain might restrict the conformational freedom of this residue by side chain-specific interactions with neighboring residues. Although the closest hydrogen bond donor, the H433 backbone amide, seemed too distant for direct interaction, a water-mediated hydrogen bond could possibly bridge this distance. Both E345 side chain rigidity and possibly associated water were therefore analyzed for several high-resolution, nonmutated Fc structures containing defined water molecules (S6A Fig). Indeed, these structures displayed limited variation in E345 carboxylic acid positioning, and approximately half contained a water molecule potentially bridging the E345 carboxylic acid group and the H433 backbone amide. E345 mutations might therefore increase conformational flexibility or potentially facilitate increased movement of H433 enabling optimized interactions with the opposing Fc.

CDC enhancement was more sidechain-specific for other stimulatory mutations at the Fc:Fc interface. I253V, S254L, Q311L, and Q311I likely contributed to optimization of the hydrophobic consensus region interaction site at the Fc:Fc interface (S6B Fig). Mutations S440Y and S440W also promoted hexamerization, possibly by stacking their aromatic moieties. However, hydrogen bonding to opposing Fc backbone atoms may also be important, given the poor efficacy of S440F (S6C Fig).

C_H_2:C_H_2 distances can vary well over 10 Å when captured by crystallization of different glycan forms [25–27]. We hypothesized that modifying the C_H_2–C_H_3 interface could lead to allosteric induction of antibody hexamerization via modulation of this dynamic conformational landscape. Functional screening identified a number of C_H_2–C_H_3 interface residues that displayed increased CDC efficacy in which residue E430 stood out (S5 Table). Remarkably, substitutions of this glutamic acid to all possible amino acids stimulated CDC, suggesting that new side chain-specific effects are unlikely explanations for the allosteric stimulation. Rather, the highly conserved salt bridge formed between C_H_3 residue E430 and C_H_2 residue K338 might limit the population of C_H_2 domains occupying a hexamerization-favorable state (S6D Fig). The disruption of the salt bridge alone, however, appeared insufficient, as modification of the pairing residue K338 only had a very limited effect on CDC (S5 Table).

The glutamic acids at positions 345 and 430 are conserved in all human IgG subclasses. Since a substitution into any other amino acid results in increased hexamerization and complement activation, it may therefore be speculated that glutamic acid at these positions may play a role in restricting exaggerated complement activation. In addition, some substitutions at these two positions increased the propensity to bind C1q in solution, as measured by a target independent C4d ELISA and native MS, and led to increased clearance from the murine circulation. The prevention of IgG self-association by E345 and E430 might thus enable natural access to a more extensive fraction of the antibody repertoire expressed as soluble IgG.

The results described therefore provide a number of amino acid positions in the antibody Fc region that enhance the ability of IgG molecules to form hexamers, which provide a docking and activation structure for avid C1 binding and efficient complement activation. The mutants E430G and E345K exemplify the preferred IgG1 backbones. These mutants enable potent CDC for a range of antibodies against different targets, while preventing target-independent complement activation in solution at therapeutically applied mAb concentrations, which are generally > 20-fold lower than natural IgG1 titers. In designing the enhanced hexamerization antibody platform, we selected backbones with optimal manufacturability and developability characteristics including criteria requiring an absence of antigen-independent complement activation. When applying the hexamerization-enhanced backbones in a novel antibody or Fc-fusion protein, a careful assessment of safety and potential efficacy will need to be performed to establish the therapeutic window, as with any new drug. For each specific disease target, it will therefore be important to determine the potential utility of enhanced IgG hexamerization as a therapeutic strategy. This potential is envisaged to be dependent on e.g., expression of antigen and its ability to multimerize, the potential downstream effects of multimerization, the expression of complement regulatory proteins on target cells, the availability of complement factors, as well as the expression of antigen on normal tissue.

The IgG1 Fc backbones identified provide a novel platform for the generation of therapeutics with enhanced effector functions that only become activated upon target-cell binding and hexamerization. When compared to protein- and glyco-engineered therapeutic antibody technologies based on increased binding to effector molecules with “always-on” characteristics, it is this conditional enhancement that uniquely differentiates this novel platform inspired by the naturally occurring hexamerization of IgG antibodies.

## Materials and Methods

### Construction, Expression, and Purification of Antibody Variants

Antibodies were expressed recombinantly in-house using an IgG1 heavy chain with the allotype G1m(f), or bought via the pharmacy (ALM, OBN). The following mAbs were used: CD20 mAbs 7D8, 11B8, RTX and OBN [16,28,29], CD38 mAbs IgG1-005 and IgG1-003 [18], EGFR mAb 2F8 [30], HIV-1 gp120 mAb IgG1-b12 [31], CD52 mAb (ALM) [32]. ALM variant E345R contained an additional K409R mutation known not to impact CDC.

Codon-optimized antibody genes encoding heavy and light chains (GeneArt, Germany) were cloned separately into pcDNA3.3 (Life Technologies, US). Mutations were introduced in heavy chain expression vectors either using Quikchange technology (Agilent Technologies, US), or via gene-synthesis (Geneart, Germany), at the indicated positions numbered according to EU nomenclature. Antibodies were expressed in HEK293 FreeStyle cells by transfection of light chain and heavy chain expression vector DNA using 293fectin essentially, as described [33]. Antibodies were purified by Protein A affinity chromatography (rProtein A FF; GE Healthcare), dialyzed overnight to PBS, and filter-sterilized over 0.2-μM dead-end filters. The concentration of purified IgGs was determined by absorbance at 280 nm. Quality assessment of purified antibodies was performed by SDS/PAGE (>90% intact IgG, >95% HC + LC under reducing conditions), ESI-TOF MS (identity confirmation), and HP-SEC (aggregate level <5%). All discussed proteins were purified and met these quality criteria unless explicitly stated otherwise.

### Construction and Expression of Fc-Mutant Antibody Library

A focused library of mutations at the positions indicated in S4 and S5 Tables was generated by site-directed mutagenesis. Mutations were introduced into the IgG1-005 Fc region using the Quikchange site-directed mutagenesis kit (Agilent, US). Briefly, for each desired mutation position, a forward and a reverse primer encoding a degenerate NNS codon at the desired location were used to replicate a full length pcDNA3.3 (Life Technologies, US) plasmid DNA template containing the IgG1-005 heavy chain with IgG1m(f) allotype. The resulting DNA mixtures were digested using DpnI to remove source plasmid DNA and used to transform *Escherichia coli*. Plasmid DNA was extracted from bacterial cultures inoculated with colonies pooled per position, and retransformed into *E*. *coli* to obtain clonal colonies.

Mutant plasmid DNA isolated from resulting individual colonies was checked by DNA sequencing (LGC genomics, Berlin, Germany). Expression cassettes were amplified from hit picked DNA plasmids by PCR and DNA mixes containing both a mutant heavy and a wild-type light chain of IgG1-005 were transiently transfected to Freestyle HEK293F cells (Invitrogen, US) using 293fectin (Invitrogen, US) essentially as described [33]. Supernatants of transfected cells containing antibody mutants were collected.

Mutant antibody supernatants were screened in CDC assays as follows. 0.1 x 10^6^ Daudi or Wien 133 cells were preincubated in round-bottom 96-well plates with 1.0 μg/ml of unpurified antibodies in a total volume of 100 μL for 15 min on a shaker at RT. As controls we included: 40 μL mock transfected HEK293 supernatants or PBS and IgG1-b12 as an isotype control antibody. Next, 30 μL normal human serum was added as a source of complement (30% final concentration) and incubated in a 37°C incubator for 45 min. The reaction was stopped by putting the plates on ice, followed by adding 10 μl propidium iodide (PI) and determination of cell lysis by FACS.

### Cell Culture and Reagents

The A431 human epidermoid cell line and DOHH-2, MEC-2, SU-DHL-4, and WSU-NHL human lymphoma cell lines were obtained from the Deutsche Sammlung von Mikroorganismen und Zellkulturen (cell line numbers ACC 91, ACC 47, ACC 500, ACC 495, and ACC 58 respectively; Braunschweig, Germany). ARH-77, Daudi, Raji, Ramos, and WIL2-S cell lines (human lymphoma) were obtained from the American Type Culture Collection (ATCC no. CRL-1621, CCL-213, CCL-86 and CRL-1596 respectively; Rockville, MD). Wien 133 cells (human Burkitt’s lymphoma) were kindly provided by Dr. Geoff Hale (BioAnaLab Limited, Oxford, UK). Raji-luc cells were generated by electroporation of Raji cells with gWIZ luciferase (Aldevron, Fargo, ND) and pPur vector (BD Biosciences, Alphen aan de Rijn, The Netherlands) in a 4:1 ratio and, after 48 h, puromycin was added for selecting a stably transfected clone (Raji-luc #2D1). Daudi, Raji, WIL2-S, ARH-77, DOHH-2, Ramos, WSU-NHL, and SU-DHL-4 were cultured in RPMI 1640 supplemented with 10% heat-inactivated CCS, 1 U/mL penicillin, 1 μg/mL streptomycin, and 4 mM L-glutamine. Raji-luc #2D1 cells were supplemented with 1 μg/mL puromicin. MEC-2 and Wien 133 were cultured in IMDM supplemented with 10% heat-inactivated FCS, 1 U/mL penicillin, and 1 μg/mL streptomycin (all media and supplements were obtained from Lonza, Vervier, Belgium). HEK293 Freestyle cells were obtained from Life Technologies (formerly Invitrogen, Paisley, UK). All cell lines were routinely tested for mycoplasma contamination and generally aliquoted and banked to allow in vitro assays to be performed from frozen cells instead of continuously cultured systems to ensure authenticity of the cell lines. Pooled normal human serum (NHS) AB was obtained from Sanquin (The Netherlands). Primary B cells from CLL patients were obtained from AllCells (Alameda, CA).

### Animals

Eight- to 11-wk-old female SCID mice (C.B-17/Icr- Prkdcscid) were obtained from Charles River Laboratories and housed in a barrier unit of the Central Laboratory Animal Facility. The mice were kept in IVC cages with water and food provided ad libitum. Mice were checked daily for clinical signs of disease and discomfort. All animal experiments were performed in compliance with the Dutch animal protection law (WoD) translated from the directives (2010/63/EU) and if applicable, the Code of Practice “animal experiments for cancer research” (Inspection V&W, Zutphen, The Netherlands, 1999). The animals were housed and handled in accordance with good animal practice as defined by FELASA, in an AAALAC and ISO 9001:2000 accredited animal facility. The local animal welfare body as well as the Animal Welfare Committee Utrecht University (DEC-Utrecht) approved all the animal experiments (2012.II.08.123 for the pharmacokinetic analysis, and 2011.III.03.036 for in vivo efficacy studies).

### ADCC

The ADCC assay was performed as described by Overdijk et al. [34]. Briefly, Raji cells were labeled with 100 μCi ^51^Cr (Amersham Biosciences, Uppsala, Sweden) and 4 h ADCC assays were performed according to standard procedures, using peripheral blood mononuclear cells (PBMC) from healthy donors as effector cells at a 100:1 effector:target ratio. The percentage NK cells was determined by staining for anti-CD56 (BD 555516, BD Biosciences, Aalst, Belgium) and analyzing on flow cytometer (FACS canto II, BD Biosciences). FcγRIIa-H131R (rs1801274) and FcγRIIIa-V158F (rs396991) genotyping in the PBMC were determined by predesigned SNP genotyping TaqMan SNP assays according to the manufacturer’s protocol (Thermo Fisher Scientific, Waltham, MA USA).

### Capillary Electrophoresis SDS (CE-SDS)

Purity and fragmentation of the samples were analyzed using CE-SDS on the Labchip GXII (Caliper Life Sciences). Sample preparation was performed in 96-well Bio-Rad HSP9601 plates using the HT Protein Express Reagent Kit according to manufacturer’s instructions (High Sensitivity protocol) with few modifications. Both nonreduced and reduced samples (addition of DTT) were prepared and denatured by incubation at 70°C for 10 min. The chip was prepared according to manufacturer’s instructions, and the samples were run with the HT antibody analysis 200 high sensitivity settings. Data were analyzed for molecular weight and purity (fraction of total) with Labchip GXII software.

### CDC Assay

CDC assays were performed as described [35] with an antibody concentration series or a fixed antibody concentration and normal human serum (20% final concentration unless indicated otherwise) as a source of complement. Killing was calculated as fraction TOPRO-iodide+ cells (%) determined by a Celigo imaging cytometer (Brooks Life Science Systems) for A431 carcinoma cells, and as the fraction PI+ cells (%) determined by a BD FACSCanto II flow cytometer for all other cells.

### Complement Activation in Normal Human Serum

Complement activation in the absence of target was determined by measuring C4d concentrations, a marker for classical complement pathway activation, after incubating 100 μg antibody per mL in 90% normal human serum for 1 h at 37°C. C4d concentrations were measured in an ELISA (MicroVue C4d EIA kit, Quidel Corporation, San Diego, US) according to the manufacturer’s instructions.

### Dynamic Light Scattering

Dynamic Light Scattering measurements were performed on a DynaPro Plate Reader II (Wyatt Technology) with Dynamics 7 software. Samples were applied in duplicate in a 384 well plate (Black, Round 384 IQ-LV, Aurora Biotechnologies, Cat no. 1011–00110) at 30 μL volume and covered with paraffin oil. Prior to the measurement the plates were centrifuged for 3 min at 2,500 xg. Samples were kept at 25°C, and 10 acquisitions of each 5 sec were recorded for each sample with auto-adjustment of attenuator and laser power. The cumulant fit procedure was used to analyze the data. A refractive index of 1.333 for PBS buffer at 20°C was used and a viscosity of 1.019 cP (standard software values supplied in Dynamics software). The apparent Rh of mutants was divided by the Rh of wild-type mAb, each averaged over three experiments.

### Hollow Fiber Flow Field Flow Fractionation

Hollow Fiber flow Field Flow Fractionation analysis was performed using an Agilent 1100 HPLC and an Eclipse DualTec system (Wyatt, US), monitoring UV at 280 nm (Agilent) and connected to a multiangle laser light scattering MiniDawn Treos detector (Wyatt, US). 4.0 μL protein samples of 1.0 mg/mL concentration were separated in 8.7 mM Na_2_HPO_4_/1.8 mM NaH_2_PO_4_/200 mM NaCl buffered at pH 7.4 in a 10 kDa PES hollow fiber with a radius of 400 μm and 17 cm length, and performed in 3-fold. Data was processed using Astra software version 6.1 (Wyatt, US). Cross-flow, focus time, elution time and flow rate optimization for IgG1-005 as well as data collection were performed by Coriolis Pharma (Martinsried, Germany).

### HP-SEC Analysis

HP-SEC fractionation was performed using a Waters Alliance 2975 separation unit (Waters, Etten-Leur, The Netherlands) connected to a TSK HP-SEC column (G3000SWxl; Toso Biosciences, via Omnilabo, Breda, The Netherlands), a Waters 2487 dual λ absorbance detector (Waters), and a Mini Dawn Treos MALS detection unit (Wyatt). 50 μL samples containing 1.25 mg/mL protein were separated at 1 mL/min in 0.1 M Na_2_SO_4_ /0.1 M sodium phosphate buffered at pH 6.8. Results were processed using Empower software version 2002 and expressed per peak as percentage of total peak area.

### Imaged Capillary Isoelectric Focusing (icIEF)

Imaged Capillary Isoelectric Focusing (icIEF) was performed using an iCE280 Analyzer (Convergent Biosciences) according to the manufacturer’s instructions. Samples were not desalted before use. Methyl Cellulose and pI markers were purchased from Convergent Bioscience, Carrier Ampholytes (Pharmalytes) from GE Healthcare. Focusing was performed for 7 min at 3,000 V, and the whole-capillary absorption image was captured by a charge-coupled device camera. After calibration of the peak profiles, the data were analyzed for pI and fractional area (%) by EZChrom software.

### Mouse Tumor Xenograft Model

5E+06 luciferase expressing Raji cells were injected subcutaneously on the right flank of 8–9 wk old, female C.B-17/IcrPrkdc-scid/CRL mice (Charles-River Laboratories, Maastricht, the Netherlands). When tumor volume reached an average of 85 mm^3^, mice were randomized and treated with a single intraperitoneal dose of 50 μg IgG1 7D8 WT, 7D8-E345R, 7D8-K322A or IgG1-b12 control antibody. Correct antibody administration was monitored by analysis of IgG levels seven days after treatment. Tumor volume was measured at least twice per week using caliper (PLEXX, Elst, The Netherlands) measurements and calculated as 0.52 x (length) x (width)^2^.

### Native MS

IgG1 oligomerization was studied by native MS as described previously [36]. The constructs were analyzed in 0.15 M ammonium acetate (pH 7.5) at an antibody concentration of 2 μM. This protein preparation was obtained by five sequential concentration and dilution steps at 4°C using a centrifugal filter with a cut-off of 10 kDa (Millipore). Samples were sprayed from borosilicate glass capillaries and analyzed on a modified LCT time-of-flight instrument (Waters, UK) adjusted for optimal performance in high mass detection [37,38]. Instrument settings were as follows; needle voltage ~1.4 kV, cone voltage ~100 V, source pressure 8.5 mbar. The extent of oligomerization was estimated by summing the areas under the curves as described previously [39].

### Photospectrometry

Antibody concentrations were determined by UV spectroscopy using a Nanodrop photospectrometer (Thermo Scientific, De Meern, Netherlands). The spectrophotometer was adjusted to baseline using PBS at 280 nm, and the absorbance of duplicate sample preparations were determined on the spectrophotometer at both 280 nm (A280) and 350 nm (A350). Sample concentrations were calculated using Beer’s law, the extinction coefficient for the specific molecule, and the A280 value.

### Pharmacokinetic Analysis

Antibodies (500 μg per mouse) were administered intravenously to groups of mice (*n* = 3), and blood samples were drawn from the submandibular or saphenous vein at 10 min, 3 h, and 1, 2, 7, 14, 21, and 28 d after administration. Blood was collected in heparin-containing vials and centrifuged (5 min at 10,000 × g) to separate plasma from cells. Plasma was transferred to a new vial and stored at −20°C. Total human IgG concentration in plasma samples was analyzed by ELISA. Plates were coated with 2 ug/mL m-anti-HuIgG (clone MH16-1 (Sanquin, The Netherlands) and plasma human IgG was detected by G-a-HuIgG-HRP (109-035-098, Jackson). Injected material was included as reference curve. AUC up to day 21 was addressed using Graphpad Prism and clearance was calculated as (Dose (mg.kg^−1^) * 1,000 / AUC).

### PCD

Daudi cells were incubated for 24 h with indicated antibody (1 μg/mL). Percentage PCD markers were determined using Annexin V (BD Biosciences cat. 556547) and cleaved Caspase 3 apoptosis Kits (BD Biosciences cat. 550914) via flow cytometry (FACS canto II, BD Biosciences) according to the manufacturer’s protocols (BD Biosciences).

### Visualization of Antibody Structure and Modeling of Mutants

Schematic views of the structure of human monoclonal IgG1 antibody b12 were based on the IgG1-b12 crystal structure deposited under PDB access code 1HZH, but with amino acids renumbered according to Eu nomenclature [40], using PyMOL version 1.5.0.4 (Schrödinger LLC). Mutant models were generated by selecting the rotamers with minimal steric clashes without subsequent energy minimization.

## Supporting Information

S1 Data Excel spreadsheet containing, in separate sheets, the underlying numerical data and statistical analyses for Figs 1–7 and S1 Fig to S5 Fig. (XLSX)

S1 Fig Structural analysis of CDC inhibiting mutations. (A) Comparison of the IgG1 Fc:Fc interaction as observed in IgG1-b12 Fc (PDB access code 1HZH, left), with rat FcRn (mid, 3FRU) and with *Staphylococcus aureus* protein A (right, 1FC2), respectively. FcRn (dark blue: large FcRn subunit p51; light blue: beta-2 microglobulin) and protein A (light blue) interactions were modeled by structurally aligning their interacting Fc with 1HZH using DaliLite [41]. (B) Zoom to IgG consensus interaction site composed of residues 252–255, 380, 428, and 433–436 (according to DeLano et al.) [21]. Residues were colored by median lysis as defined in Fig 3B, >60% (blue), 30%–60% (orange), or <30% (red). Two views rotated 90 degrees along the *y*-axis are indicated. (C) Zoom to the Fc:Fc interface C-terminal β-strand interaction region. Mutants of all residues 436–440 displayed a median lysis below 30% (defined in Fig 3B; S4 Table).
(EPS)

S2 Fig Validation of CDC potentiation by IgG1 Fc-domain mutations. (A) CDC of Daudi cells by wild-type and mutant IgG1-005 antibodies. A representative of three replicate experiments is shown. (B) CDC of Daudi cells by wild-type and mutant RTX antibodies. A representative of three replicate experiments is shown.
(EPS)

S3 Fig PK analysis of RTX mutants in SCID mice using three mice per group. Clearance of a single 500 μg dose of mAb was monitored for 3 wk and is expressed as 1,000 * Dose / AUC; dashed line: average clearance of wild-type RTX.
(EPS)

S4 Fig Size-distribution analysis of selected IgG mutants. (A) HP-SEC profiles of Protein A-purified IgG1-005 and mutant antibodies. Left panel: chromatograms staggered at 0.5 AU per trace along *y*-axis. Right panel: 50x zoomed chromatograms, staggered at 0.01 AU per trace. Main peaks at 9.0–9.1 min indicate monomeric species. Aggregate levels for the mutants (≤1.2%) were similar to the wild-type (<1%). (B) Hollow fiber flow field flow fractionation analysis of wild-type IgG1-005 and mutant antibodies. The triple mutant IgG1-005-RGY was used as a control for the detection of hexameric species.
(EPS)

S5 Fig Real time stability study of 100 mg/mL 7D8 and mutant antibody solutions formulated in PBS pH 7.4 incubated for three months at 5°C. (A) HP-SEC to determine the fraction of degradation products as a percentage of total protein. Detection limit of 1% is indicated by a dashed line. (B) Nonreducing CE-SDS analysis for the fraction of intact IgG as a percentage of total protein. (C) Reducing CE-SDS analysis for the fraction HC+LC as a percentage of total protein. (D) Capillary isoelectric focusing analysis for the fraction of acidic species as a percentage of total protein. (E) Capillary isoelectric focusing analysis for the fraction of basic species as a percentage of total protein. Detection limit of 2.5% is indicated by a dashed line. (F) Photospectrometric analysis at 280 nm. (G) Photospectrometric analysis at 350 nm after 100-fold dilution in PBS.
(EPS)

S6 Fig Structural analysis of CDC-regulating hotspots E345 and E430. (A) CH3 domain-based structural alignment of IgG1 antibody b12 (1HZH chain K, light green) with high resolution IgG Fc crystal structures, zoomed to E345. Arrow indicates oxygen atoms of E345-proximal water molecules, displayed as spheres. Dark green: 1HZH chain H; chocolate: 4KU1 [25]; dark red: 1H3U [27]; dark pink: 1H3X [27]; dark salmon: 3AY4 [42]; light pink: 3V7M [43]; purple: 4B7I [44]. (B) Molecular zoom of residues at the consensus interaction site of antibody IgG1-b12 (PDB access code 1HZH). Residues from two interacting Fc-domains are indicated: one Fc in light green ribbon with side chains indicated as sticks, the other Fc’ in light blue surface presentation, residues labeled with a prime. Noncarbon atoms were colored by element (oxygen, red; nitrogen, dark blue). (C) Molecular zoom of residues in the C-terminal β–strands of two interacting Fc-domains (green and blue, respectively) of antibody IgG1-b12. Mutant models of S440Y (mid) and S440W (right) were generated by selecting the rotamer with minimal steric hindrance, without further energy minimization. (D) Structural alignment as defined in (A), zoomed to the E430 (CH3)–K338 (CH2) salt bridge. Note the variation in CH2 domain positioning relative to the CH3 domain, captured by different crystal isoforms.
(EPS)

S1 Table EC_50_ (antibody concentration inducing half-maximal lysis) values for CDC of antibody-opsonized cells. Mean EC_50_ and SD for CDC of different cell lines opsonized with wild-type or E345R-mutated antibody and incubated in the presence of human complement were determined. Numbers of replicates and statistics are shown. (1) Number of experiments. (2) Mean and (3) standard deviation (SD) were calculated from all experiments. (4) Statistics: unpaired *t* test two tailed on log-transformed data (GraphPad Prism 5.01). Significance was calculated in comparison to the wild-type IgG1 counterpart: (n.a.) not applicable; (n.s.) not significant. (5) EC50 indicated as >μg/mL as lysis did not reach 50%. (6) Since EC50 could not be determined, upper bound of significance was calculated using maximally tested concentration as lower bound for EC50.
(DOCX)

S2 Table QiFi analysis of cell surface marker expression. Expression levels of CD20, CD38, CD52, EGFR, and the mCRPs CD46, CD55, CD59 on cell lines used in this study; n.d.: not determined. The cell lines are sorted for decreasing CD20:mCRP ratio. Numbers indicate multiples of thousand molecules per cell.
(DOCX)

S3 Table Analysis of mouse tumor xenograft growth. Top panel: tumor size monitored by caliper measurements was used to calculate average tumor size per group. At day 22, the last day at which all groups were still complete, a nonparametric Mann Whitney analysis was applied to tumor volumes of the different treatment groups using GraphPad Prism. The hexamerization-enhanced antibody 7D8-E345R inhibited tumor growth significantly when compared to the isotype control antibody IgG1-b12 and the complement-deficient mutant 7D8-K322A. Bottom panel: Time to progression (cut-off set at tumor volume >700 mm^3^) was analyzed by a Mantel-Cox pairwise comparison test using SPSS. When compared to IgG1-b12 control antibody, only 7D8-345R antibody-inhibited tumor progression significantly.
(DOCX)

S4 Table IgG1-005 Fc domain mutant library CDC screen using Daudi cells at 1.0 μg/mL mutant IgG1. The condition was chosen as it gives high CDC (>80%) for the wild-type antibody and therefore provides for a screening condition in which inhibition of complement activation by specific mutations can be assessed. Numbers indicate percentage cell lysis. Lysis and SD of control antibodies is summarized below the main table; total number of control replicates is indicated in brackets. Controls include: mock transfected HEK293 supernatants, PBS and IgG1-b12 as an isotype control antibody.
(DOCX)

S5 Table IgG1-005 Fc domain mutant library CDC screen using Wien 133 cells at 1.0 μg/mL mutant IgG1. The condition was chosen as it gives low CDC (<15%) for the wild-type antibody and provides for a screening condition in which enhancement of complement activation by specific mutations can be assessed. Numbers indicate percentage cell lysis. Lysis and SD of control antibodies is summarized below the main table. The total number of control replicates is indicated in brackets. Controls include: mock transfected HEK293 supernatants, PBS, and IgG1-b12 as an isotype control antibody.
(DOCX)

S6 Table EC_50_ (antibody concentration inducing half-maximal lysis) values for CDC of IgG1-005 antibody variant opsonized cells. Mean EC_50_ and SD for CDC of Daudi cells opsonized with wild-type or mutant IgG1-005 and incubated in the presence of human complement were determined. Numbers of replicates and statistics are shown. (1) Number of experiments. (2) Mean and SD were calculated from all experiments. (3) One-way ANOVA on log-transformed data followed by Dunnett's Multiple Comparison Posthoc Test using GraphPad Prism 6.04. Significance was calculated in comparison to the wild-type IgG1-005; (n.a.) not applicable.
(DOCX)

S7 Table EC_50_ (antibody concentration inducing half-maximal lysis) values for CDC of RTX antibody variant opsonized cells. Mean EC_50_ and SD for CDC of Daudi cells opsonized with wild-type or mutant RTX and incubated in the presence of human complement were determined. Numbers of replicates and statistics are shown. (1) Number of experiments. (2) Mean and SD were calculated from all experiments. (3) One-way ANOVA on log-transformed data followed by Dunnett's Multiple Comparison Posthoc Test using GraphPad Prism 6.04. Significance was calculated in comparison to the wild-type RTX; (n.a.) not applicable.
(DOCX)

## Abbreviations

ADCC: antibody dependent cellular cytotoxicity
ALM: alemtuzumab
AUC: area under the curve
CDC: complement-dependent cytotoxicity
CLL: chronic lymphocytic leukemia
DLS: dynamic light scattering
EGFR: epidermal growth factor receptor
HAG: heat aggregated IgG
HP-SEC: high-performance size exclusion chromatography
mAbs: monoclonal antibodies
MACs: membrane attack complexes
mCRP: membrane complement regulatory protein
MS: mass spectrometry
Mw: molecular weight
OBN: obinutuzumab
PCD: programmed cell death
PI: propidium iodide
Rh: hydrodynamic radius
RTX: rituximab
SCID: severe combined immunodeficiency
SD: standard deviation
SEM: standard error of the mean
