# Supplementary material for: A Novel Platform for the Potentiation of Therapeutic Antibodies Based on Antigen-Dependent Formation of IgG Hexamers at the Cell Surface
Source: PLoS Biol. 2016 Jan 6;14(1):e1002344. doi: 10.1371/journal.pbio.1002344 (PMC4703389; doi:10.1371/journal.pbio.1002344)
Supplement: S1 Table — Mean EC50 and SD for CDC of different cell lines opsonized with wild-type or E345R-mutated antibody and incubated in the presence of human complement were determined. Numbers of replicates and statistics are shown. (1) Number of experiments. (2) Mean and (3) standard deviation (SD) were calculated from all experiments. (4) Statistics: unpaired t test two tailed on log-transformed data (GraphPad Prism 5.01). Significance was calculated in comparison to the wild-type IgG1 counterpart: (n.a.) not applicable; (n.s.) not significant. (5) EC50 indicated as >μg/mL as lysis did not reach 50%. (6) Since EC50 could not be determined, upper bound of significance was calculated using maximally tested concentration as lower bound for EC50. (DOCX) [file pbio.1002344.s008.docx]

S1 Table. EC_50_ (antibody concentration inducing half-maximal lysis) values for CDC of antibody-opsonized cells.

| **Antibody** | **Cell line** | ***N^(^*^1)^** | **Mean EC50 (µg/mL)^(2)^** | **SD^(3)^** | **Significance^(4)^** |
| --- | --- | --- | --- | --- | --- |
| RTX | Wien133 | 4 | 0.90 | 0.64 |  |
| RTX-E345R | Wien133 | 4 | 0.25 | 0.17 | n.s. |
| 11B8 | Wien133 | 6 | >10 |  |  |
| 11B8-E345R | Wien133 | 6 | 0.16 | 0.18 | P < 0.001^6^ |
| RTX | Daudi | 4 | 1.82 | 0.75 |  |
| RTX-E345R | Daudi | 4 | 0.19 | 0.10 | P < 0.01 |
| 11B8 | Daudi | 5 | >10 |  |  |
| 11B8-E345R | Daudi | 5 | 0.07 | 0.05 | P < 0.001^6^ |
| ALM | Wien 133 | 3 | 2.05 | 0.35 |  |
| ALM-E345R | Wien 133 | 3 | 0.04 | 0.01 | P < 0.0001 |
| ALM | Raji | 3 | >30 |  |  |
| ALM-E345R | Raji | 3 | 1.35 | 0.43 | P < 0.001^6^ |
| IgG1-003 | Wien133 | 4 | >3 |  |  |
| IgG1-003-E345R | Wien133 | 4 | 0.39 | 0.23 | P < 0.001^6^ |
| IgG1-005 | Wien133 | 3 | >3 |  |  |
| IgG1-005-E345R | Wien133 | 3 | 0.03 | 0.03 | P < 0.001^6^ |
| IgG1-003 | Daudi | 4 | >3 |  |  |
| IgG1-003-E345R | Daudi | 4 | 0.35 | 0.26 | P < 0.001^6^ |
| IgG1-005 | Daudi | 3 | 0.17 | 0.09 |  |
| IgG1-005-E345R | Daudi | 3 | 0.02 | 0.01 | P < 0.01 |
| 2F8 | A431 | 3 | >10 |  |  |
| 2F8-E345R | A431 | 3 | 0.13 | 0.04 | P < 0.001^6^ |
