# Supplementary material for: A Novel Platform for the Potentiation of Therapeutic Antibodies Based on Antigen-Dependent Formation of IgG Hexamers at the Cell Surface
Source: PLoS Biol. 2016 Jan 6;14(1):e1002344. doi: 10.1371/journal.pbio.1002344 (PMC4703389; doi:10.1371/journal.pbio.1002344)
Supplement: S2 Table — Expression levels of CD20, CD38, CD52, EGFR, and the mCRPs CD46, CD55, CD59 on cell lines used in this study; n.d.: not determined. The cell lines are sorted for decreasing CD20:mCRP ratio. Numbers indicate multiples of thousand molecules per cell. (DOCX) [file pbio.1002344.s009.docx]

S2 Table. QiFi analysis of cell surface marker expression.

| **Cell Line** | **Targets** | | | |  | **mCRP** | | | | $\frac{\begin{aligned} \\ \mathbf{CD20} \end{aligned}}{\mathbf{mCRP}}$ |
| --- | --- | --- | --- | --- | --- | --- | --- | --- | --- | --- |
|  | **CD20** | **CD38** | **CD52** | **EGFR** |  | **CD46** | **CD55** | **CD59** | **total** |  |
| **WIL2-S** | 244±47 | 0 | 4±0.2 | n.d. |  | 11±0.6 | 1.6±0.2 | 0.5±0.2 | 13.1 | 18.6 |
| **Wien 133** | 137±25 | 26±6 | 72±11 | n.d. |  | 12±0.2 | 1.7±0.2 | 2.6±0.4 | 16.3 | 8.4 |
| **Daudi** | 279±41 | 199±18 | 2±0 | n.d. |  | 29±5 | 5±3 | 4±1 | 38 | 7.3 |
| **SU-DHL4** | 672±228 | 146±13 | n.d. | n.d. |  | 50±4 | 18±2 | 69±6 | 137 | 4.9 |
| **DOHH-2** | 352±64 | 147±28 | 8±0.2 | n.d. |  | 54±4 | 33±3 | 79±13 | 166 | 2.1 |
| **Raji** | 134±15 | 170±48 | 15±3 | n.d. |  | 49±10 | 18±3 | 6±2 | 73 | 1.8 |
| **WSU-NHL** | 256±50 | 48±7 | n.d. | n.d. |  | 67±4 | 9±2 | 145±19 | 221 | 1.2 |
| **ARH-77** | 265±67 | 26±3 | n.d. | n.d. |  | 64±6 | 34±1 | 179±33 | 277 | 1.0 |
| **MEC-2** | 264±32 | 95±5 | 40±5 | n.d. |  | 63±5 | 30±3 | 163±24 | 256 | 1.0 |
|  |  |  |  |  |  |  |  |  |  |  |
| **A431** | n.d. | n.d. | n.d. | 1202±574 |  | 148±49 | 16±1 | 319±32 | 483 | n.a. |
