# Supplementary material for: A Novel Platform for the Potentiation of Therapeutic Antibodies Based on Antigen-Dependent Formation of IgG Hexamers at the Cell Surface
Source: PLoS Biol. 2016 Jan 6;14(1):e1002344. doi: 10.1371/journal.pbio.1002344 (PMC4703389; doi:10.1371/journal.pbio.1002344)
Supplement: S3 Table — Top panel: tumor size monitored by caliper measurements was used to calculate average tumor size per group. At day 22, the last day at which all groups were still complete, a nonparametric Mann Whitney analysis was applied to tumor volumes of the different treatment groups using GraphPad Prism. The hexamerization-enhanced antibody 7D8-E345R inhibited tumor growth significantly when compared to the isotype control antibody IgG1-b12 and the complement-deficient mutant 7D8-K322A. Bottom panel: Time to progression (cut-off set at tumor volume >700 mm3) was analyzed by a Mantel-Cox pairwise comparison test using SPSS. When compared to IgG1-b12 control antibody, only 7D8-345R antibody-inhibited tumor progression significantly. (DOCX) [file pbio.1002344.s010.docx]

S3 Table. Analysis of mouse tumor xenograft growth.

| **Mann Whitney test** | **IgG1-b12** | **IgG1-b12** | **IgG1-b12** | **7D8-K322A** | **7D8** |
| --- | --- | --- | --- | --- | --- |
| **at day 22** | **vs.** | **vs.** | **vs.** | **vs.** | **vs.** |
|  | **7D8-E345R** | **7D8-K322A** | **7D8** | **7D8-E345R** | **7D8-K322A** |
| P value | 0.0044 | 0.3799 | 0.1441 | 0.0474 | 0.4688 |
| P value summary | ** | ns | ns | * | ns |
| Significantly different?  (p < 0.05) | Yes | No | No | Yes | No |
| One- or two-tailed P value? | Two-tailed | Two-tailed | Two-tailed | Two-tailed | Two-tailed |
| *Difference between medians* |  |  |  |  |  |
| Median of column A | 157.0, n=9 | 368.0, n=9 | 289.5, n=8 | 157.0, n=9 | 368.0, n=9 |
| Median of column D | 533.0, n=9 | 533.0, n=9 | 533.0, n=9 | 368.0, n=9 | 289.5, n=8 |
| Difference: Actual | 376 | 165 | 243.5 | 211 | -78.5 |
| Difference: Hodges-Lehmann | 365 | 129 | 218.5 | 211 | -85 |

| Log Rank (Mantel-Cox) P-value | IgG1-b12 | 7D8 | 7D8-K322A | 7D8-E345R |
| --- | --- | --- | --- | --- |
| IgG1-b12 | - |  |  |  |
| 7D8 | 0.175 | - |  |  |
| 7D8-K322A | 0.387 | 0.729 | - |  |
| 7D8-E345R | 0.019 | 0.144 | 0.085 | - |
