# Supplementary material for: A Novel Platform for the Potentiation of Therapeutic Antibodies Based on Antigen-Dependent Formation of IgG Hexamers at the Cell Surface
Source: PLoS Biol. 2016 Jan 6;14(1):e1002344. doi: 10.1371/journal.pbio.1002344 (PMC4703389; doi:10.1371/journal.pbio.1002344)
Supplement: S5 Table — The condition was chosen as it gives low CDC (<15%) for the wild-type antibody and provides for a screening condition in which enhancement of complement activation by specific mutations can be assessed. Numbers indicate percentage cell lysis. Lysis and SD of control antibodies is summarized below the main table. The total number of control replicates is indicated in brackets. Controls include: mock transfected HEK293 supernatants, PBS, and IgG1-b12 as an isotype control antibody. (DOCX) [file pbio.1002344.s012.docx]

S5 Table. IgG1-005 Fc domain mutant library CDC screen using Wien 133 cells at 1.0 µg/mL mutant IgG1.

| **Amino acid** | **Location** | **A** | **C** | **D** | **E** | **F** | **G** | **H** | **I** | **K** | **L** | **M** | **N** | **P** | **Q** | **R** | **S** | **T** | **V** | **W** | **Y** |
| --- | --- | --- | --- | --- | --- | --- | --- | --- | --- | --- | --- | --- | --- | --- | --- | --- | --- | --- | --- | --- | --- |
| **P247** | CH2:CH3 | **5** | **5** | **16** |  | **16** | **54** | **11** | **19** | **14** | **4** | **17** | **7** | **5** |  | **10** | **12** | **4** | **3** | **9** |  |
| **M252** | Fc:Fc | **3** |  |  |  |  | **4** | **4** | **7** |  |  |  | **3** | **4** |  | **3** | **5** |  | **24** | **5** |  |
| **I253** | Fc:Fc | **11** |  | **9** |  |  |  |  |  | **3** |  | **3** | **3** |  |  | **4** | **3** |  | **46** |  |  |
| **S254** | Fc:Fc |  |  |  | **14** | **16** | **32** | **2** | **15** | **12** | **67** |  |  | **2** |  |  |  | **9** |  | **9** |  |
| **T256** | Fc:Fc |  |  | **3** |  |  |  |  |  |  | **3** | **23** | **5** |  | **7** |  | **3** |  |  |  | **11** |
| **T359** | Fc:Fc |  |  |  |  |  | **2** |  |  |  |  |  | **3** | **4** |  | **41** |  |  |  |  |  |
| **L309** | Fc:Fc |  |  |  |  | **15** | **3** |  |  |  |  |  |  |  |  | **2** | **3** |  | **2** |  |  |
| **H310** | Fc:Fc | **6** |  |  |  | **7** | **4** |  |  | **3** | **3** |  |  | **17** |  | **5** |  | **3** | **7** | **18** |  |
| **Q311** | CH2:CH3 | **9** | **4** |  | **3** | **21** | **4** | **6** | **58** | **18** | **68** |  | **6** | **20** |  | **29** | **9** | **3** |  | **40** | **19** |
| **K338** | CH2:CH3 | **2** | **2** | **2** |  |  | **5** |  |  |  |  |  |  | **33** | **8** | **5** | **16** |  | **2** |  |  |
| **A339** | Fc:Fc |  |  |  |  | **4** | **4** |  |  | **17** |  |  | **35** | **25** | **13** | **6** |  |  |  |  |  |
| **E345** | Fc:Fc | **73** | **52** | **74** |  | **66** | **68** | **68** | **71** | **80** | **79** | **75** | **78** | **72** | **95** | **83** | **73** | **69** | **78** | **71** | **83** |
| **E356** | Fc:Fc |  |  |  |  |  | **28** |  | **25** |  | **27** |  |  |  |  | **69** |  | **27** | **13** |  |  |
| **P374** | CH2:CH3 |  |  |  | **26** |  | **2** |  |  | **13** | **15** |  |  |  |  | **18** | **2** |  | **12** | **12** | **20** |
| **D376** | CH2:CH3 |  |  |  |  | **7** |  |  |  | **2** | **8** |  | **10** | **2** |  |  |  |  |  |  | **4** |
| **A378** | CH2:CH3 |  |  | **2** |  |  |  |  |  | **2** | **2** |  |  | **2** |  | **7** | **2** |  |  |  | **7** |
| **E380** | Fc:Fc |  |  |  |  | **5** |  |  | **3** | **2** | **3** |  | **5** |  | **2** | **3** |  | **3** | **2** | **2** | **11** |
| **E382** | Fc:Fc |  |  |  |  | **2** |  |  |  | **2** | **41** | **19** |  | **3** |  |  |  |  | **43** | **2** |  |
| **N384** | Fc:Fc | **4** | **3** |  | **4** | **4** |  |  | **4** |  |  |  |  | **2** |  | **5** | **4** | **3** | **4** | **5** |  |
| **G385** | Fc:Fc | **5** |  | **5** |  |  |  | **4** |  |  | **4** |  | **23** | **5** | **4** | **12** | **4** | **4** | **5** |  |  |
| **Q386** | Fc:Fc | **3** | **4** | **4** | **4** | **3** | **3** | **3** | **4** | **69** | **3** |  | **4** | **2** |  | **4** | **3** | **7** | **3** | **3** | **4** |
| **P387** | Fc:Fc |  |  |  |  | **2** | **2** | **4** |  |  | **2** |  |  |  |  | **2** | **3** | **3** |  |  |  |
| **V422** | Fc:Fc | **3** |  |  | **2** |  |  |  | **3** |  | **3** |  | **2** |  |  | **2** | **4** |  |  |  |  |
| **M428** | Fc:Fc |  |  |  |  | **3** | **3** | **2** |  | **2** | **2** |  | **5** | **37** | **6** | **3** |  | **2** |  |  |  |
| **E430** | CH2:CH3 | **70** | **84** | **90** |  | **85** | **82** | **80** | **95** | **47** | **80** | **69** | **95** | **80** | **63** | **70** | **61** | **97** | **72** | **97** | **97** |
| **N434** | Fc:Fc |  |  | **2** | **2** |  | **2** |  |  | **2** |  |  |  |  |  | **2** | **3** |  | **2** | **18** |  |
| **Y436** | Fc:Fc |  |  |  |  |  |  |  | **51** | **3** | **4** |  |  |  |  | **3** | **3** | **2** |  | **3** |  |
| **T437** | Fc:Fc |  | **2** |  |  | **2** | **4** |  |  | **2** |  |  |  | **2** |  |  |  |  | **2** |  |  |
| **Q438** | Fc:Fc |  | **15** |  | **3** |  |  |  | **3** | **3** | **10** |  |  |  |  |  | **2** | **2** | **2** | **2** | **2** |
| **K439** | Fc:Fc | **3** |  | **2** | **2** |  |  | **2** |  |  | **2** |  |  | **2** |  |  |  | **2** |  |  | **4** |
| **S440** | Fc:Fc | **8** | **6** | **31** | **7** | **7** | **7** | **28** | **6** | **3** | **6** | **8** | **6** | **10** | **18** | **6** |  | **6** | **8** | **89** | **80** |
| **P445** | Fc:Fc | **3** | **5** | **2** | **2** | **4** | **2** | **4** |  | **4** | **3** |  |  |  |  |  |  | **2** | **3** |  |  |
| **G446** | Fc:Fc |  |  |  |  | **3** |  |  |  | **4** | **5** | **4** |  |  |  | **7** |  |  | **3** | **7** |  |
| **K447** | Fc:Fc |  |  |  |  |  | **3** |  | **4** |  | **3** |  |  | **3** |  | **2** |  |  | **2** | **6** |  |

| **Control Ab** | **IgG1-005** | **005-E345R** | **005-K439E** | **005-S440K** | **b12** | **Mock** | **no Ab** |
| --- | --- | --- | --- | --- | --- | --- | --- |
| Lysis ± SD  (# replicates) | 11.0±7.1  (46) | 83.4±15.2  (51) | 2.2±0.3  (5) | 2.3±0.4  (5) | 7.7±9.7  (7) | 7.0±5.1  (10) | 2.5±0.5  (10) |
