# Supplementary material for: A Novel Platform for the Potentiation of Therapeutic Antibodies Based on Antigen-Dependent Formation of IgG Hexamers at the Cell Surface
Source: PLoS Biol. 2016 Jan 6;14(1):e1002344. doi: 10.1371/journal.pbio.1002344 (PMC4703389; doi:10.1371/journal.pbio.1002344)
Supplement: S7 Table — Mean EC50 and SD for CDC of Daudi cells opsonized with wild-type or mutant RTX and incubated in the presence of human complement were determined. Numbers of replicates and statistics are shown. (1) Number of experiments. (2) Mean and SD were calculated from all experiments. (3) One-way ANOVA on log-transformed data followed by Dunnett's Multiple Comparison Posthoc Test using GraphPad Prism 6.04. Significance was calculated in comparison to the wild-type RTX; (n.a.) not applicable. (DOCX) [file pbio.1002344.s014.docx]

S7 Table. EC_50_ (antibody concentration inducing half-maximal lysis) values for CDC of RTX antibody variant opsonized cells.

| Antibody | *N*^(1)^ | Mean EC_50_  (µg/mL)^(2)^ | SD^(2)^ | Significance^(3)^ |
| --- | --- | --- | --- | --- |
| RTX | 5 | 1.3 | 0.71 | n.a. |
| RTX-E345K | 3 | 0.089 | 0.049 | p≤0.0001 |
| RTX-E345Q | 2 | 0.088 | 0.065 | p≤0.0001 |
| RTX-E345R | 5 | 0.044 | 0.027 | p≤0.0001 |
| RTX-E345Y | 2 | 0.077 | 0.054 | p≤0.0001 |
| RTX-E430G | 4 | 0.086 | 0.019 | p≤0.0001 |
| RTX-E430H | 2 | 0.081 | 0.045 | p≤0.0001 |
| RTX-E430S | 2 | 0.15 | 0.095 | p≤0.001 |
| RTX-E430T | 4 | 0.071 | 0.011 | p≤0.0001 |
| RTX-S440Y | 4 | 0.16 | 0.032 | p≤0.0001 |
